# High molecular weight, but not total, CTRP3 levels are associated with serum triglyceride levels

**DOI:** 10.14814/phy2.14306

**Published:** 2019-12-09

**Authors:** Greta Trogen, Arsham Alamian, Jonathan M. Peterson

**Affiliations:** ^1^ East Tennessee State University Johnson City Tennessee; ^2^ Department of Biostatistics and Epidemiology College of Public Health East Tennessee State University Johnson City Tennessee; ^3^ Department of Health Sciences College of Public Health East Tennessee State, University Johnson City Tennessee; ^4^ Quillen College of Medicine Department of Biomedical Sciences East Tennessee State University Johnson City Tennessee

**Keywords:** adipokines, adiponectin, adipose tissue‐derived cytokine, CTRP3

## Abstract

C1q/TNF‐related protein 3 (CTRP3) is a relatively novel adipose tissue‐derived cytokine (adipokine) which has been linked to improved glucose regulation and insulin sensitivity. However, the relationship between circulating CTRP3 levels and diabetes is controversial. CTRP3 can circulate in different oligomeric complexes: trimeric, hexameric, and high molecular weight (HMW) oligomeric complexes. However, the concentration of the different oligomeric complexes in human disease states has not been previously investigated. Therefore, the purpose of this study was to compare the levels of different oligomeric complexes of CTRP3 between type 2 diabetic and nondiabetic individuals. Additionally, the association between the oligomeric complexes and other serum factors was examined. CTRP3 primarily circulates in the HMW complex (>50%) and the hexametric multimer, with no CTRP3 detected in the trimeric complex or as a monomer. Further, no differences were observed in total, hexameric, or HMW CTRP3 levels regardless of diabetic status. Surprisingly, HMW CTRP3 was found to be positively correlated with circulating triglyceride levels. Combined, these data suggest that CTRP3 is associated with triglyceride regulation, not diabetic status. These data may explain some of the discrepancies in the literature as elevated triglyceride levels are often detected in patients with obesity and type 2 diabetes.

## Graphical abstract

CTRP3 can circulate in different oligomeric complexes: trimeric, hexameric, and high molecular weight (HMW) oligomeric complexes. This study found that increased levels of HMW oligomeric complex of CTRP3 were associated with higher circulating triglycerides.

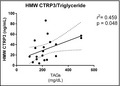

## 1 INTRODUCTION

The C1q/TNF superfamily contains an array of secreted proteins that contribute to organismal homeostasis through a number of diverse and sometimes opposing effects on processes such as host defense, inflammation, development, cell differentiation, and metabolic processes (Kishore et al., 2004; Shapiro & Scherer, 1998). C1q/TNF‐related protein 3 (CTRP3) is a highly conserved member of the C1q/TNF superfamily, containing the characteristic C‐terminal globular domain and N‐terminal collagenous repeats (Gly‐X‐Y) (Peterson, Seldin, Wei, Aja, & Wong, 2013; Peterson, Wei, & Wong, 2010; Wong et al., 2008). CTRP3 was originally identified as CORS26 (collagenous repeat‐containing sequence 26 kDa protein) and is also known as cartducin/cartonectin due to its expression in developing cartilage (Maeda, Abe, Kurisu, Jikko, & Furukawa, 2001; Maeda et al., 2006; Schaffler, Weigert, Neumeier, Scholmerich, & Buechler, 2007). However, in adults, CTRP3 expression is primarily found in adipose tissue indicating that, at least in adults, it is an adipokine (Wong et al., 2008). Further, CTRP3 has been shown to be a potent insulin sensitizer and inhibits inflammation (Peterson et al., 2010), indicating that CTRP3 could play a major role in metabolic diseases such as obesity, nonalcoholic fatty liver disease, and type 2 diabetes. However, the associations between total CTRP3 levels with obesity or metabolic syndrome are controversial, as circulating CTRP3 levels have been shown to be elevated, decreased, or not changed with obesity (Li, Wright, & Peterson, 2017). These discrepancies indicate that the role of CTRP3 in human health is more complicated than can be identified by total circulating levels.

Adiponectin/Acrp30 (adipocyte complement‐related protein of 30 kDa, hereafter referred to as adiponectin) is the most widely studied adipokine of the C1q/TNF superfamily. Adiponectin is present in human serum as three distinct multimeric complexes: trimeric low molecular weight (LMW), hexameric medium molecular weight (MMW), and oligomeric high molecular weight (HMW) complexes containing 12–18 proteins (Pajvani et al., 2003). Previous studies have found that the inability to form the larger adiponectin protein was associated with diabetes (Arita et al., 1999; Waki et al., 2003), even if total adiponectin levels were the same, indicating that MMW or LMW oligomeric versions were not functional. An association between the size of adiponectin and metabolic dysfunction (such as diabetes and obesity) has been repeatedly observed in the literature as reviewed by Hirose, Yamamoto, Seino‐Yoshihara, Kawabe, and Saito (2010) in 2010. Similar to adiponectin, CTRP3 is secreted in the HMW, MMW, and LMW oligomeric states (Wong et al., 2008); however, the relationship between the oligomeric states of circulating CTRP3 and metabolic disease has not been explored. Therefore, the purpose of this study is to analyze the oligomeric state of circulating CTRP3 in relation to the presence or absence of severe metabolic disease (i.e., type 2 diabetes). The primary hypothesis was that, although total levels of circulating CTRP3 will be similar between people with type 2 diabetes compared with nondiabetic controls, there will be a difference between groups in the circulating oligomeric state of CTRP3. The secondary purpose of this study was to determine whether the circulating oligomeric state of CTRP3 correlates with the circulating levels of inflammatory cytokines or triglycerides as CTRP3 has been previously demonstrated to attenuate inflammation and to reduce triglyceride levels (Hofmann et al., 2011; Kopp, Bala, Buechler, et al., 2010; Kopp, Bala, Weigert, et al., 2010; Li et al., 2017; Petersen et al., 2016; Peterson et al., 2013, 2010; Schmid, Kopp, Hanses, Karrasch, & Schaffler, 2014; Tan et al., 2013; Trogen et al., 2018; Yang et al., 2016; Yoo et al., 2013).

## 2 METHODS

### 2.1 Samples

De‐identified age‐matched type 2 diabetic and nondiabetic human plasma samples were obtained from Physicians Plasma Alliance (https://www.pparesearch.com/product/type-2-diabetes-plasma). Type 2 diabetes status was verified by clinician and the exclusion criteria were current glucose‐lowering drug use, uncontrolled hypertension (SBP > 160 mmHg or DBP> 100 mmHg), history of cardiovascular disease defined as stable coronary artery disease or acute coronary syndrome, stroke or transient ischemic attack, or peripheral artery disease. Age, sex, body mass index (BMI), and HbA1c levels were indicated for each sample.

### 2.2 Plasma analysis

All assays were performed according to the manufacturer's directions except when specifically indicated. Inflammatory cytokines (IL‐6, IL‐8, IL‐10, and TNF‐α) were quantified using a commercial cytokine multiplex assay (Bio‐Rad Cat# M50000007A). Total plasma triglyceride levels were determined by Thermo Scientific™ Triglycerides Reagent (Cat# TR22421). CTRP3 levels were quantified by commercial ELISA (R&D Systems ELISA; DY7925‐05). Specific assay working ranges, coefficients of variation, and limits of detection are listed in Table 1. Any value below the detectable assay working range was assigned the value equivalent to the lower limit of quantification.

**Table 1:** Assay working ranges

	Assay working ranges		Assay precision
	Lower limit of quantification	Upper limit of quantification	Intra‐assay (%CV)
IL‐6 (pg/ml)	0.5	8,412	1.3
IL‐8 (pg/ml)	1.1	16,849	4.1
IL‐10 (pg/ml)	1.9	25,112	1.4
TNF (pg/ml)	3.1	57,207	1.3
Triglycerides (mg/dl)	62.5	1,000	3.6
CTRP3 (ng/ml)	20	450	13.4

Total circulating CTRP3 levels were determined according to the manufacturer's directions. However, to determine the changes of CTRP3 oligomer composition, samples were first separated into fraction using a series of size‐exclusion filtration. Briefly, the HMW fractions were concentrated using a 300 kDa size‐exclusion centrifugal concentrator (Sartorius™ Vivaspin™; VS0151). The filtrate containing the MMW and LMW fractions was then concentrated using the 100 kDa filtration tube size‐exclusion centrifugal concentrator (Sartorius™ Vivaspin™; VS0142). The final filtrate now containing only the LMW fractions (trimer ~ 70–90 Kda and monomer ~30 kDa) was then collected. Each fraction was re‐diluted to the starting volume and CTRP3 levels were quantified using the R&D Systems ELISA (DY7925‐05) and reported as ng/ml.

### 2.3 Statistical analysis

Descriptive statistics (mean and standard deviation) were calculated for all measured variables. An unpaired two‐tailed *t* test was used to compare means between diabetic and nondiabetic groups. Pearson correlation coefficient was performed to measure the correlation between the serum variables and total CTRP3 as well as CTRP3 oligomer levels. All statistical analyses were performed by Graphpad Prism 6.

## 3 RESULTS

### 3.1 Subject characteristics

Characteristics of subjects are presented in Table 2. Briefly, plasma samples represented 20 unique individuals of a similar age range and the HbA1c levels were greater than 9% in the type 2 diabetic and below 6% in the nondiabetic individuals.

**Table 2:** Subject characteristics

	Control	Type 2 diabetic
Sex (male/female)	8/2	5/5
Age (years; mean, SD)	45.7 ± 14.2	44.6 ± 5.1
BMI (kg/m^2^; mean, SD)	31.6 ± 6.6	39.43 ± 8.8*
HbA1c (%; mean, SD)	5.42 ± 0.25	10.66 ± 0.80*

Abbreviations: BMI, body mass index; HbA1c, glycated hemoglobin.

* *p* < .05, unpaired two‐tailed *t* test.

### 3.2 Difference in circulating levels between groups

**Figure 1:**
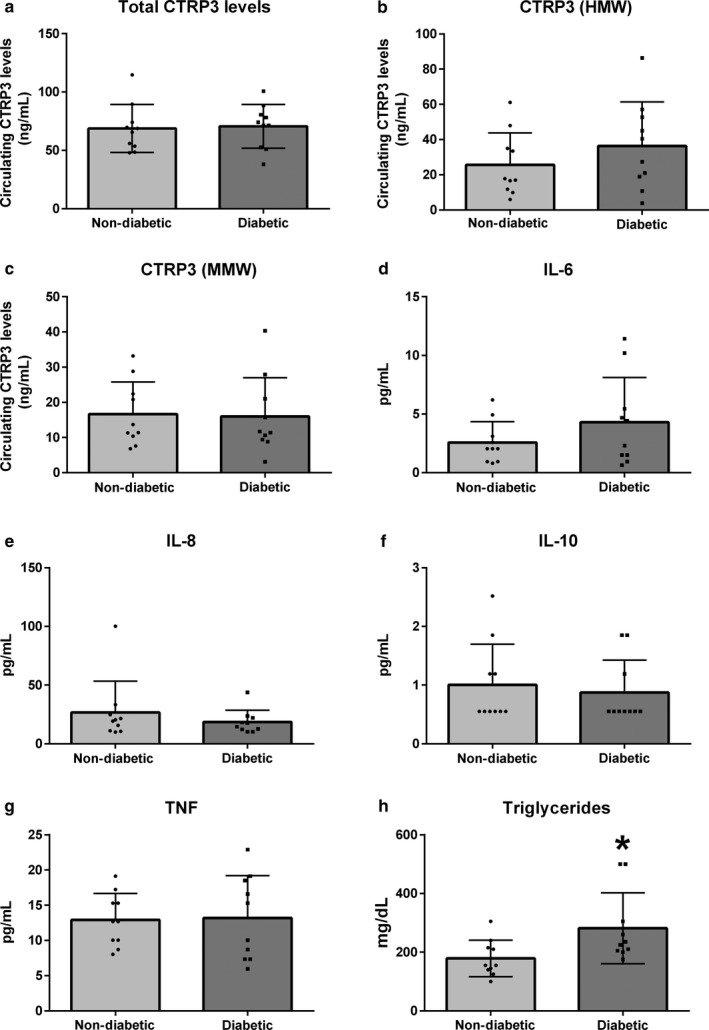
Levels of total CTRP3 (a), HMW CTRP3 (b), MMW CTRP3 (c), IL‐6 (d), IL‐8 (e), IL‐10 (d), TNF (G), and triglycerides were measured in plasma samples from diabetic and healthy control samples. Mean and standard deviations are reported with black dots representing individual values. *p < .05 between nondiabetic and diabetic groups as calculated by unpaired two‐tailed *t* test. HMW, high molecular weight oligomeric complex; MMW, hexameric medium molecular weight; IL, interleukin; TNF, tumor necrosis factor alpha

The average values for all measured analytes in samples are presented in Figure 1. Briefly, total, HMW, and MMW CTRP3 levels were similar between nondiabetic and diabetic groups (Figure 1a–c). Surprisingly, LMW CTRP3 was not detected in any sample measured, indicating that CTRP3 primarily circulates in the MMW and HMW oligomers in humans. Analysis of serum cytokines found no difference between groups in IL‐6, IL‐8, IL‐10, or TNF values (Figure 1d–h). As expected the diabetic groups had a significant elevation in serum triglyceride levels, ~40% higher in the diabetic group compared to the healthy group (mean ± *SD*, 281.5 ± 38.1 mg/dl vs. 179.0 ± 19.7, respectively, *p* = .02).

### 3.3 Correlations of CTRP3 concentrations and triglyceride levels

**Figure 2:**
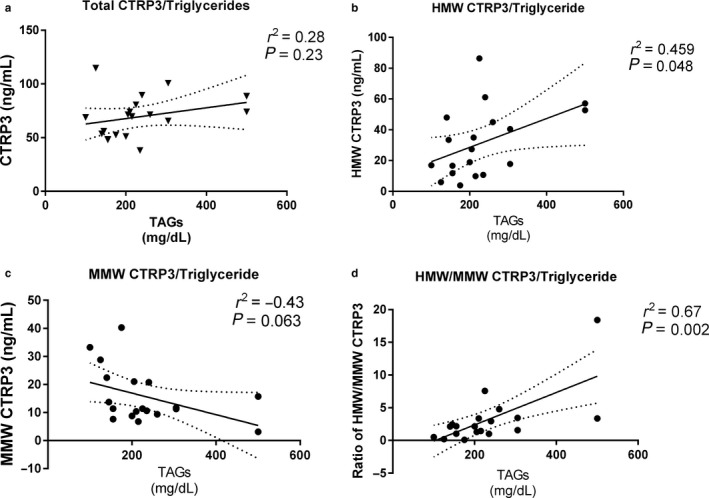
Correlations between circulating CTRP3 and triglyceride levels. Pearson correlation coefficient was calculated to determine the correlation between circulating triglyceride levels and total CTRP3 as well as the HMW and MMW oligomers of CTRP3. Black dots represent individual samples. HMW, high molecular weight oligomeric complex; MMW, hexameric medium molecular weight

In order to examine the biological mechanisms for CTRP3, correlations between the total and oligomeric conformations of CTRP3 and all other measured variables were tested by Pearson's correlation analysis (Table 3). As presented in Figure 2, total and MMW CTRP3 concentrations were not correlated with any value measured, although the negative correlation between MMW CTRP3 and triglyceride levels as well as total CTRP3 and BMI both approached significance (*p* = .06 and *p* = .07, respectively). HMW CTRP3 was significantly positively correlated with serum triglyceride levels (Figure 2b; *r* ^2^ = .46, *p* = .048). Further, the ratio of HMW CTRP3 to MMW CTRP3 had an even stronger significant positive correlation with circulating triglyceride levels (Figure 2d; *r* ^2^ = .67, *p* = .002).

**Table 3:** Pearson's correlation analysis of CTRP3

	Age (years)	Sex	IL‐6 (pg/ml)	IL‐8 (pg/ml)	IL‐10 (pg/ml)	TNF (pg/ml)	HbA1c	TAGs (mg/dl)
Total CTRP3	0.76	0.88	0.69	0.01	0.43	0.94	0.92	0.243
HMW	0.42	0.11	0.13	0.26	0.42	0.54	0.63	0.048*
MMW	0.33	0.36	0.26	0.09	0.22	0.79	0.89	0.063
HMW/MMW	0.55	0.07	0.29	0.52	0.42	0.73	0.25	0.002*

Abbreviations: HMW, high molecular weight oligomeric complex of CTRP3; MMW, hexameric medium molecular weight of CTRP3.

* *p* < .05, Pearson's correlation analysis.

## 4 DISCUSSION

This is the first study to examine the circulating oligomeric state of CTRP3 in a disease state. The major significant finding of this study is that almost all circulating CTRP3 in humans is found as either the MMW or HMW oligomer. This finding supports previous speculation that the oligomer conformation of CTRP3 is important for its functional activity (Li et al., 2017; Peterson, 2016; Peterson et al., 2010). The secondary finding of this study is that total CTRP3 levels were almost identical between diabetic and nondiabetic populations (68.8 ± 20 vs. 70.7 ± 18 ng/ml), and there was no difference between these groups in circulating concentration of HMW or MMW CTRP3. These data do not support our initial hypothesis that diabetic status would result in differences in the circulating multimer distribution of CTRP3.

Wagner et al. previously reported that total CTRP3 levels are not correlated with circulating triglyceride levels in a group of patients (*n* = 100) with coronary artery disease symptoms (Wagner, Sivagnanam, Clark, & Peterson, 2016), and this finding was supported in this study population. However, contrary to the findings of this study, Tan et al. (2013) reported a significant negative correlation between total CTRP3 and circulating triglyceride levels in a large group (*n* = 122) consisting of solely of females, with or without polycystic ovary syndrome (PCOS). Further, Wolf et al. (2015) also reported a negative correlation between total CTRP3 and circulating triglyceride levels in obese patients (*n* = 44), that was not present in lean patients (*n* = 60). These data indicate that CTRP3 is associated with triglyceride regulation, but clearly demonstrate that the understanding of the regulation of CTRP3 and its relationship with circulating triglycerides is not fully elucidated. In an attempt to explore the role of CTRP3 further, this study is the first to examine the multimer distribution of CTRP3, which has demonstrated a significant positive correlation of HMW CTRP3 as well as HMW/MMW CTRP3 ratio to circulating triglyceride levels. These novel findings support the hypothesis that CTRP3 contributes to triglyceride regulation, and indicate that the role of the different oligomeric conformations of CTRP3 and triglyceride regulation need to be explored further.

Transgenic overexpression of CTRP3 has been shown to prevent both alcoholic and nonalcoholic fatty liver disease in rodent studies (Peterson et al., 2013; Trogen et al., 2018). CTRP3 also reduces hepatic triglyceride synthesis and secretion, further supporting the role of CTRP3 in triglyceride regulation (Peterson et al., 2013). However, research in human subjects has been equivocal, with total circulating CTRP3 levels reported to be reduced (Ban et al., 2014; Deng et al., 2015; Qu et al., 2015; Tan et al., 2013; Wolf et al., 2015) or elevated (Choi et al., 2014, 2012) with metabolic conditions such as obesity and/or metabolic syndrome. Further complicating the understanding between the relationship of CTRP3 and obesity is that CTRP3 was reported to be elevated with obesity in males but decreased with obesity in females (Wagner et al., (2016). Interestingly, circulating CTRP3 levels are reduced in women with polycystic ovarian syndrome, but restored with metformin treatment (Tan et al., 2013). It is important to note that metformin is not only an insulin sensitizer, but also lowers circulating triglyceride levels (Geerling et al., 2014). Overall, these data support the findings of this study that CTRP3 is involved with triglyceride regulation. However, the role of CTRP3 is likely linked to the specific multimeric distributions within a given population.

## 5 CONCLUSION

The findings of this study do not support our original hypothesis that CTRP3 (regardless of its oligomeric state) is protective against diabetes. However, the correlation between HMW/hexameric CTRP3 and triglyceride levels suggests that CTRP3 contributes to triglyceride regulation, but is dependent on different oligomeric complexes and requires further study.

### 5.1 Study limitations

The small sample size limits the significance of the study findings. However, this manuscript is the first to examine the different oligomer conformations of CTRP3 found in human circulation, and documents the absence of circulating trimeric CTRP3. The relatively small sample size does increase the chance for a type I error, especially with regard to HMW CTRP3, as the standard deviation was approximately 70% of the group average. The group size selected was determined to provide sufficient power (*β* = .08) to detect differences in mean total CTRP3 levels greater than 1.5 standard deviation of the group mean.

Another major limitation of this study is that although the groups were matched by age, they were not matched by BMI or sex. While both groups had lean and obese subjects, the diabetic group had a significantly higher BMI and had more females. Our previous research has shown a divergence between the sexes in the regulation of total CTRP3 levels with obesity (Wagner et al., 2016), and the small sample size of this study prevents subdividing the data further. Therefore, the relationship between the triglyceride levels and multimeric distribution of CTRP3 should be repeated in a larger group within each sex independently to confirm these findings.

Lastly, the cross‐sectional nature of this study precludes making causal claims. However, combined with the previous findings of CTRP3 preventing hepatic triglyceride synthesis in rodents, a potential mechanism for CTRP3‐induced triglyceride regulation has been identified and requires further analysis.

## CONFLICT OF INTEREST

None declared.
